# Effect of varicella‐zoster virus infection and antiviral treatment on the risk for dementia: A meta‐analysis of observational studies

**DOI:** 10.1002/brb3.3407

**Published:** 2024-02-05

**Authors:** Li‐fei Zhu, Chuan‐cai Hu, Yu Mei, Min‐jing Zhu, Ting‐yun Ye

**Affiliations:** ^1^ Department of Clinical Laboratory Sanmen People's Hospital Taizhou Zhejiang China; ^2^ Clinical Lab Traditional Chinese Medicine of Sanmen Renming Hospital Taizhou Zhejiang China

**Keywords:** Alzheimer disease, evidence‐based, herpes virus, infection, virus

## Abstract

**Background and purpose:**

There is increasing evidence to support a role for human herpes viruses in the development of neurodegenerative disorders; however, the association between varicella‐zoster virus (VZV) infection and dementia, and the effect of antiviral therapy on the risk for dementia remain unclear.

**Methods:**

We searched PubMed, Embase, and the Cochrane Library databases from their dates of inception to May 2023. Odds ratios (ORs) with 95% confidence intervals (CIs) served as indicators of effect sizes in evaluations of the effect of VZV infection and antiviral treatment on dementia risk. Subgroup analyses based on study design, study location, diagnostic criteria of dementia, and VZV subtype classification were also performed.

**Results:**

A total of 10 studies with 316,846 dementia cases were included in the meta‐analysis. We found that any VZV infection (OR: 1.04, 95% CI: .97–1.12; *p* = .22), herpes zoster (HZ) (OR: 1.05, 95% CI: .96–1.13; *p* = .28), or HZ involving the cranial nerves (OR: 1.36, 95% CI: .76–2.43; *p*  =  .304) was not associated with an increased risk of dementia. The results of subgroup analyses were consistent with these findings. However, patients infected with VZV who received antiviral treatment had a lower OR than untreated patients infected with VZV. Compared to individuals not infected with VZV, antiviral therapy in those infected with VZV was associated with reduced risk for dementia.

**Conclusion:**

The association between VZV infection and dementia may be masked by antiviral treatment. Further studies with longer follow‐up times that consider the severity of VZV infection and antiviral treatment are needed to clarify the contribution of VZV infection to the risk for dementia.

## Abstract 2

This systematic review and meta‐analysis provide evidence of the effect of VZV infection and antiviral treatment on the risk for dementia. The findings suggest that the association between VZV infection and dementia may be masked by antiviral treatment. Further studies that take antiviral treatment into consideration are needed to establish this actual association.

## 1 INTRODUCTION

Primary infection with varicella‐zoster virus (VZV) is usually confined to children and is typically characterized by a self‐limiting fever and chickenpox (Heininger & Seward, 2006). After the resolution of the primary infection, VZV enters a lifelong latent state in the sensory ganglia (Gershon et al., 2015). Reactivation of latent VZV infection during a decline in cell‐mediated immunity causes herpes zoster (HZ), which is characterized by painful unilateral vesicles or blisters with a typical dermatomal distribution (Steiner et al., 2007). The incidence tends to increase with advancing age and rises sharply after the age of 50 years (Yawn & Gilden, 2013). In the context of population aging, HZ has become an important public health problem.

Dementia is a progressive neurodegenerative disease that often manifests as memory deficits and cognitive decline (Livingston et al., 2020). Among individuals >65 years of age, 8.1% are estimated to suffer from dementia, which severely threatens families and societies. Many efforts have been made over the past decade to identify infectious pathogens that can cause dementia (Ou et al., 2020). Recently, an association between VZV infection and dementia has been suggested. Several cases of cognitive impairment or dementia onset after VZV encephalitis have been reported (Bangen et al., 2010; Grahn et al., 2013). Thus, epidemiological studies have been conducted, but the results have been mixed. In the first such work, HZ ophthalmicus was reported to strongly increase the risk for dementia during a 5‐year follow‐up period (Tsai et al., 2017). However, a recent systematic review summarized data regarding the association between human herpes virus infections and dementia and reported that VZV infection does not increase the risk for dementia (Warren‐Gash et al., 2019). In the time since that review was published, at least 7 studies (Choi et al., 2021; Johannesdottir Schmidt et al., 2022; Lopatko Lindman et al., 2021; Lophatananon et al., 2021; Schnier et al., 2021; Shim et al., 2022; Warren‐Gash et al., 2022) with 6 million participants have focused on a possible relationship between VZV infection and dementia. Although most of those studies evaluated the relationship between VZV infection and the dementia risk, a few also examined whether the risk for dementia after VZV infection might be modified by antiviral treatment status. However, the effect of VZV infection and antiviral treatment on the risk for dementia is still unclear. We therefore conducted a meta‐analysis to examine this relationship, based on all observational studies published up to May 2023.

## 2 METHODS

### 2.1 Literature search

This meta‐analysis adhered to the Preferred Reporting Items of Systematic Reviews and Meta‐Analyses guidelines. In May 2023, we searched the PubMed, Embase, and Cochrane Register of Controlled Trials databases. The search strategies were database‐specific; we used the following keywords: (zoster OR shingles OR zona OR HZ OR antiherpetic) AND (dementia OR Alzheimer OR frontotemporal dementia OR cognitive dysfunction OR cognitive impair OR cognitive decline OR vascular dementia OR multi‐infarct dementia OR neurodegenerative diseases OR neurocognitive disorders). We also reviewed the references cited by all retrieved articles.

### 2.2 Inclusion and exclusion criteria

The eligibility criteria were (1) observational studies (case–control or cohort studies) evaluating the effect of VZV infection or antiviral treatment on the risk for dementia; (2) a focus on VZV infection status; (3) a focus on dementia as the outcome; (4) presentation of multivariate‐adjusted risk ratios (ORs), relative risks, or hazard ratios with 95% confidence intervals (CIs); and (5) sample size >1000. We excluded articles that were not in English, animal and genetic studies, case reports and series, editorials, correspondence, conference abstracts, and reviews.

### 2.3 Data extraction

Two authors independently extracted first author names, publication years, the countries and settings, and the study designs and periods; they also extracted patient characteristics (ages at baseline, sample sizes, classification of VZV infection type, antiviral treatment, dementia assessment methods, and follow‐up durations). Any disagreements were resolved by consultation with a third reviewer.

### 2.4 Outcome assessment

The primary analysis focused on assessing the risk for dementia after VZV infection. In an effort to explain the observed among‐study heterogeneity, subgroup analyses were performed based on study design (case–control vs. cohort), location (Asia vs. Europe), type classification of VZV infection (VZV infection vs. HZ vs. HZ ophthalmicus), type of dementia (Alzheimer's disease vs. vascular dementia vs. other), dementia assessment method (diagnostic code vs. diagnostic code or a prescription of an anti‐dementia drug), and age at entry (50–59 vs. 60–69 vs. ≥70 years). The secondary analysis was performed to evaluate the modified effect of antiviral treatment on the risk for dementia. Antiviral treatment was defined as the prescription of any of the following medications: acyclovir, cidofovir, famciclovir, ganciclovir, valacyclovir, or valganciclovir.

Dementia subtypes were divided into three categories according to the definitions used in the included studies: (1) Alzheimer's disease, (2) vascular dementia, (3) other: frontotemporal dementia, dementia with Lewy bodies, Parkinson's disease dementia, alcoholic dementia, and others.

VZV infections were divided into three types of classifications: (1) VZV infection: seropositivity or DNA of VZV or VZV manifestation; (2) symptomatic zoster: HZ; (3) symptomatic zoster: HZ involving the cranial nerves.

### 2.5 Quality assessment

Two reviewers independently used the nine‐item Newcastle–Ottawa scale to assess study quality (Higgins, 2014). This scale rates the quality of an observational study in three domains: selection (four questions) and comparability (two questions) of the study groups, along with the exposure (case–control) or outcome (cohort) of interest (three questions); all items are scored 0 or 1. The scale assigns a maximum of 9 points; studies with Newcastle–Ottawa scale scores ≥7 were considered high quality.

### 2.6 Statistical analysis

STATA software ver. 11.0 (Stata Corporation) was used for all analyses. The *I* ^2^ statistic was used to evaluate heterogeneity; *I* ^2^  > 50% was considered indicative of significant heterogeneity (Higgins & Thompson, 2002). The data were pooled using the DerSimonian and Laird random effects models to manage study heterogeneity (Higgins et al., 2003). ORs were regarded as approximations of relative risks or hazard ratios because dementia was rare in all study populations. Splitting one study into several estimates allowed substantially more weight to be assigned to that study in the meta‐analysis, especially in the random effects model. Thus, if more than three estimates from one study were included, a fixed effects model was employed to generate a pooled OR, which was then used in the meta‐analysis. Forest plots were used to display the effect sizes and the pooled results. The Egger test (rather than the Begg test) was used to assess publication bias because the number of included studies was small (Egger et al., 1997; Lau et al., 2006). All statistical tests were two‐sided; *p*‐values <.05 were considered indicative of statistical significance.

## 3 RESULTS

### 3.1 Search results

**FIGURE 1:**
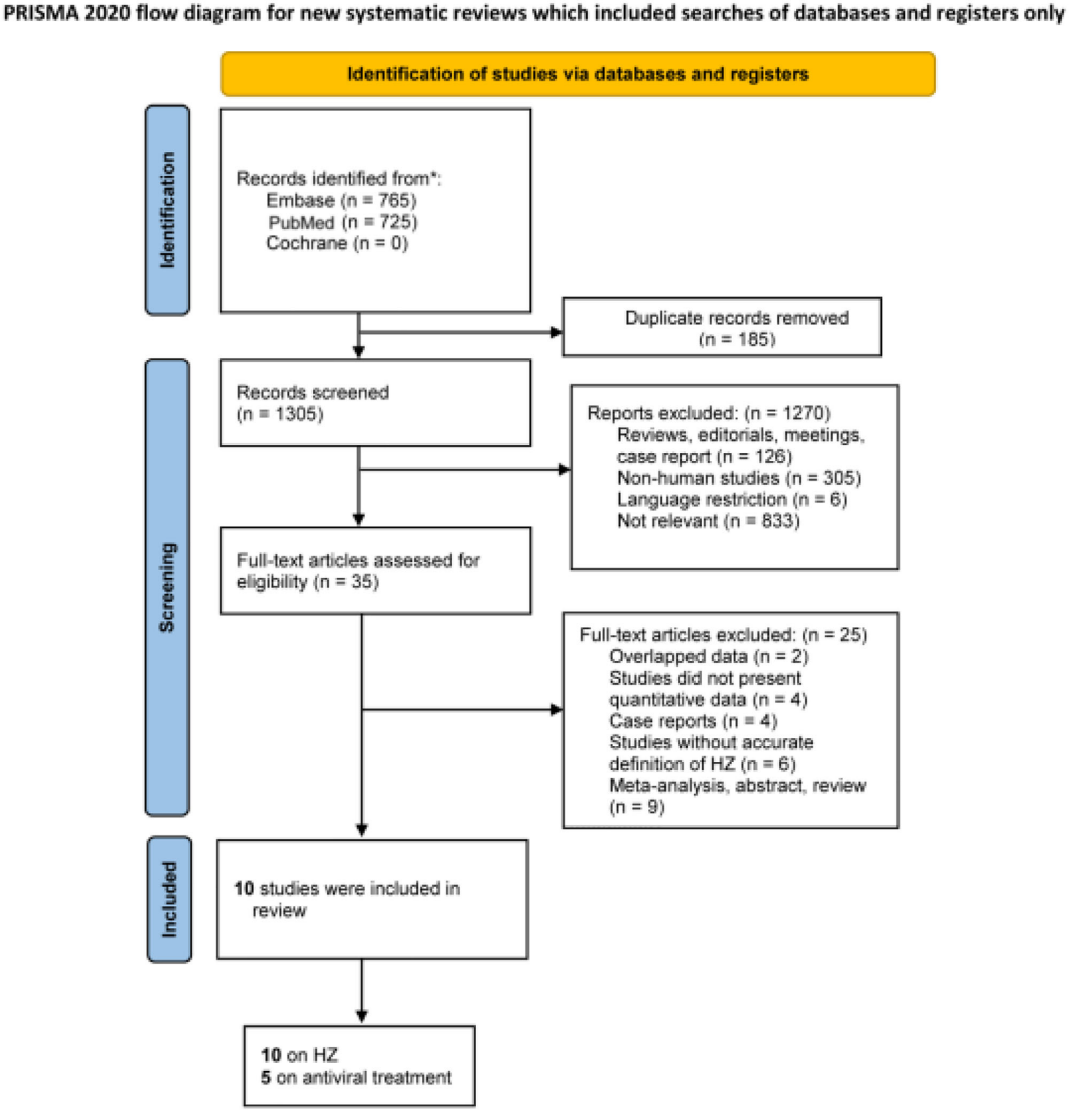
Flow chart of the search process and study selection.

The search flow is presented in a PRISMA diagram (Figure 1). After the removal of 185 duplicates, our initial search yielded 1305 studies; a total of 1270 of these studies were excluded after title and abstract screening, and 35 studies underwent full‐text review. Three studies that used Korean National Health Insurance Service databases were included in this systematic review because they were conducted by different investigators and differed in their design and inclusion criteria, although there was some overlap of study periods and participants. Therefore, a sensitivity analysis was performed to assess the influence of each study on heterogeneity, and a summary estimate was obtained by including each of these studies from the same database one at a time. Ultimately, 10 studies (Bae et al., 2021; Chen et al., 2018; Choi et al., 2021; Johannesdottir Schmidt et al., 2022; Lopatko Lindman et al., 2021; Lophatananon et al., 2021; Schnier et al., 2021; Shim et al., 2022; Tsai et al., 2017; Warren‐Gash et al., 2022) were included in our analysis.

### 3.2 Characteristics of the included studies

The included studies are summarized in Table 1. All 10 studies were population‐based; eight were cohort studies, and two were case–control studies. The studies were conducted in Korea (three), Taiwan (two), the United Kingdom (two), and Sweden and Denmark (one). The remaining study was conducted using data from Wales, Germany, Scotland, and Denmark. Among the included studies, eight evaluated the association between HZ and dementia. Two studies explored the relationship between VZV infection and dementia, but they did not classify VZV infection according to subtype. Five studies explored the relationship between antiviral treatment and dementia. The sample size ranged from 3384 to 2543,055, with a total of 3780,218 participants. The number of dementia cases per study ranged from 81 to 129,662, with an overall total of 316,846. The study quality ranged from seven points (moderate) to nine points (high), with a mean of 8.3 points; thus, the overall quality was sufficient to allow meta‐analysis. Tables S1 and S2 list the Newcastle–Ottawa scale score details.

**TABLE 1:** Characteristics of the included studies.

Reference	Location, setting	Study design/period	Age (year)	Classification of VZV infection	VZV diagnostic criteria	Dementia diagnostic criteria	Sample size	Assessment antiviral on dementia risk	Duration of follow‐up (year)	Adjustment	NOS score
Tsai et al. (2017)	Taiwan, population‐based	Cohort/2001–2013	≥40	HZ involving the cranial nerves	ICD‐9‐CM	ICD‐9‐CM	HZO 846 no‐HZO 2538	No	5	Age, monthly income, geographical region, urbanization level, hypertension, diabetes, hyperlipidemia, coronary heart disease, and stroke	8
Chen et al. (2018)	Taiwan, population‐based	Cohort/1997–2013	≥50	HZ	ICD‐9‐CM	ICD‐9‐CM	HZ 39,205 no‐HZ 39,205	Yes	6.22	Sex, age, residence, depression, autoimmune disease, ischemic stroke, traumatic brain injury, alcohol use disorder, and antiviral treatments for herpes zoster	9
Bae et al. (2021)	Korea, population‐based	Cohort/2002–2013	≥50	HZ	ICD‐10	ICD‐10	HZ 35,017 no‐HZ 195,089	Yes	11	Age, sex, economic class, hypertension, diabetes, dyslipidemia, chronic lung disease, ischemic heart disease, stroke, heart failure, atrial fibrillation, valvular heart disease, chronic renal disease, carotid artery stenosis, peripheral vascular disease, chronic liver disease, rheumatic disease, inflammatory bowel disease, malignancy including hematologic malignancy and solid tumor, solid organ transplant, HIV infection, and depression	9
Choi et al. (2021)	Korea, population‐based	Case–control/2002–2013	≥60	HZ	ICD‐10	ICD‐10 and 2 prescriptions of an anti‐dementia drug	Case 11,445 Control 45,780	No	NA	Age, sex, income, region of residence, hypertension, diabetes mellitus, and dyslipidemia	7
Lophatananon et al. (2021)	The United Kingdom, population based	nested case–control/2006–2020	≥44	HZ	ICD‐10, ICD‐9, or primary care record linkage	ICD‐9 or ICD‐10	Case 2378 Control 225,845	No	>3	Age and sex	8
Lopatko Lindman et al. (2021)	Sweden, population‐based	Cohort/2005–2017	≥50	Not specified	ICD‐10	ICD‐10 or a prescription of an anti‐dementia drug	VZV 39,526 no‐VZV 29,593	Yes	>10	Sex, age, baseline comorbidity, and educational level	8
Schnier et al. (2021)	Wales, Germany, Scotland, and Denmark, population‐based	Cohort/1995–2017	≥65	HZ	Diagnostic code or a prescription of an antiviral	Diagnostic code or a prescription of an anti‐dementia drug	HZ 51,082 no‐HZ 414,687	Yes	2.7–9	Calendar year and age	8
Johannesdottir Schmidt et al. (2022)	Denmark, population‐based	Cohort/1997–2017	≥40	HZ or HZ involving the cranial nerves	ICD‐10 and antiviral treatment	ICD‐10 or a prescription of an anti‐dementia drug	HZ 247,305 no‐HZ 1,235,890	No	>10	Age, sex, autoimmune disease, chronic kidney disease, chronic obstructive pulmonary disease, asthma, hematological cancer, solid cancer, diabetes, glucocorticoids, HIV, lipid‐lowering therapy, and traumatic head injury	9
Shim et al. (2022)	Korea, population‐based	Cohort/2010–2018	≥50	Not specified	ICD‐10	ICD‐10	VZV 97,323 no‐VZV 183,779	No	10	Female sex, older age, and comorbidities such as hypertension, diabetes mellitus, hyperlipidemia, and previous stroke	9
Warren‐Gash et al. (2022)	The United Kingdom, population‐based	Cohort/2000–2017	≥40	HZ or HZ involving the cranial nerves	Diagnostic and referral codes	Diagnostic and referral codes	HZ 177,144 no‐HZ 706,901	Yes	>4	Sex, age, practice, year of study entry, frailty index, prior consultation rate, harmful alcohol use, BMI, smoking, chronic kidney disease, asthma, autoimmune disease, COPD, depression, hypertension, ischemic heart disease, severe immunosuppression, liver disease, stroke, traumatic brain injury, herpes simplex, and uncontrolled diabetes	8

Abbreviations: HZ, herpes zoster; VZV, varicella zoster virus.

### 3.3 Meta‐analysis

#### 3.3.1 Primary analysis

**FIGURE 2:**
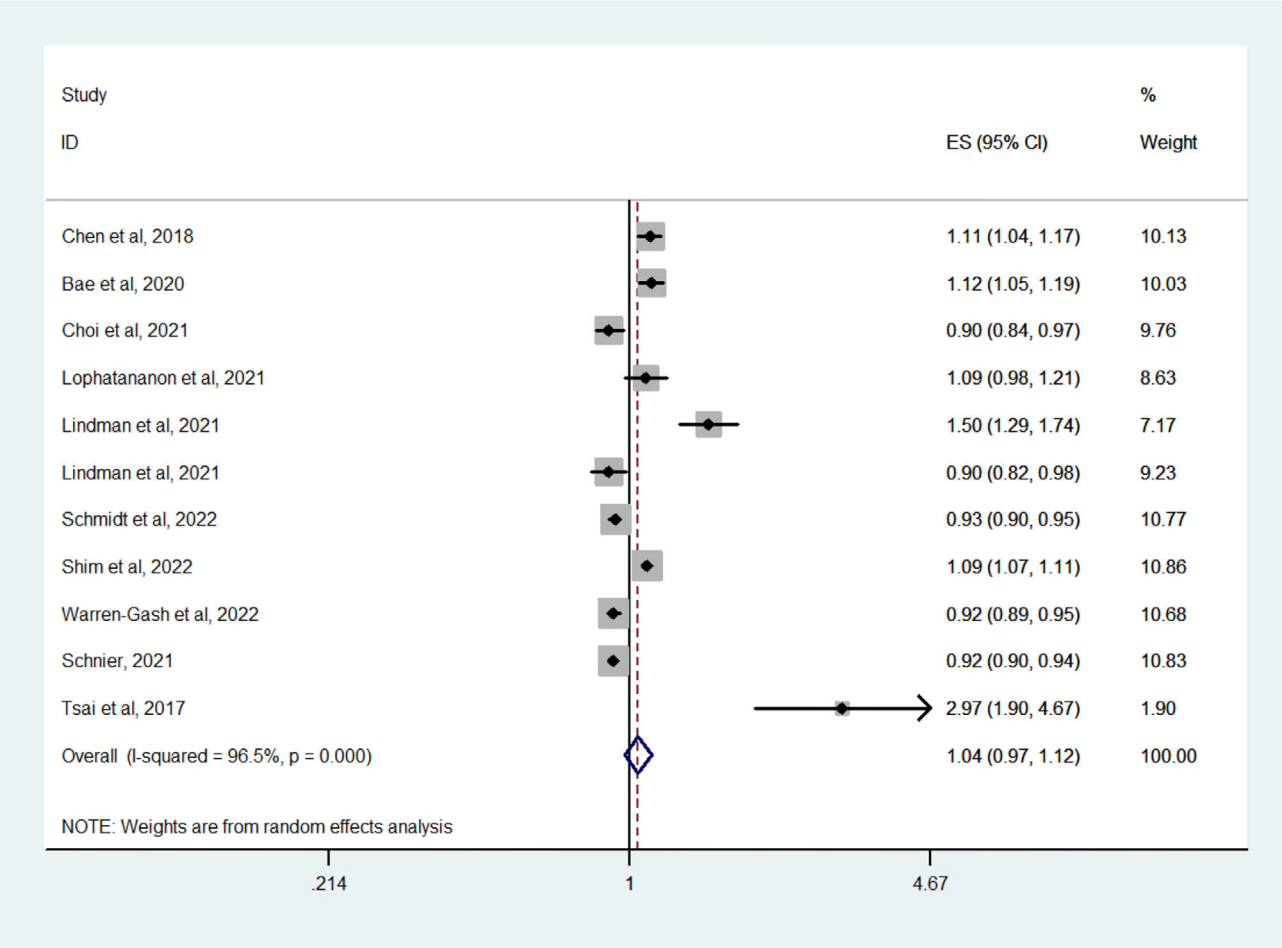
Forest plot of the overall risk of dementia in relation to varicella zoster virus (VZV) infection.

The results are summarized in Table 2. We did not find a significant association between VZV infection and dementia incidence (OR: 1.04, 95% CI: .97–1.12; *p* = .22) (Figure 2). Study heterogeneity was assessed using the *I* ^2^ statistic; significant heterogeneity (*I* ^2^ = 96.6%) was evident. Evaluation of publication bias using the Egger test (Figure S1) revealed no such bias (Egger test *p* = .6). The sensitivity analysis showed no substantial change in pooled risk estimates upon the exclusion of any single study. When the studies were grouped according to design, no significant association was found in either case–control studies (OR: 0.99, 95% CI: .82–1.19; *p* = .88) or cohort studies (OR: 1.06, 95% CI: .98–1.14; *p* = .15). When the studies were grouped according to location, there was no significant association in studies from Asia (OR: 1.09, 95% CI: 1–1.2; *p* = .058) or Europe (OR: 0.98, 95% CI: .93–1.04; *p* = .48). When the studies were grouped according to dementia measurement, no significant association was found in studies using diagnostic codes (OR: 1.1, 95% CI: 1–1.21; *p* = .054) or in studies using diagnostic codes or a prescription of an anti‐dementia drug (OR: 0.97, 95% CI: .9–1.03; *p* = .326).

**TABLE 2:** Meta‐analysis for studies included in the analysis.

Analysis	No. of studies	No. of estimates	No. of people	Pooled OR (95% CI), *I* ^2^ statistics (%)	Model used
VZV infection	10	10	3,780,578	1.04 (.97–1.12); *I* ^2^ = 96.5%	Random effects
Study design					
Case–control	2	2	269,261	0.99 (.82–1.19); *I* ^2^ = 88%	Random effects
Cohort	8	9	3511,317	1.06 (.98–1.14); *I* ^2^ = 97.2%	Random effects
Study location					
Asia	5	5	650,227	1.09 (1–1.2); *I* ^2^ = 91.5%	Random effects
Europe	5	6	3130,351	0.98 (.93–1.04); *I* ^2^ = 89.9%	Random effects
Dementia measurement					
Diagnostic code	6	6	527,027	1.1 (1–1.21); *I* ^2^ = 90.5%	Random effects
Diagnostic code or a prescription of an anti‐dementia drug	4	5	3235,551	0.97 (.9–1.03); *I* ^2^ = 92.5%	Random effects
HZ	8	8	3430,357	1.05 (.96–1.13); *I* ^2^ = 96.2%	Random effects
Study design					
Case–control	2	2	269,261	0.99 (.82–1.19); *I* ^2^ = 88%	Random effects
Cohort	6	6	3161,096	1.07 (.97–1.17); *I* ^2^ = 97.1%	Random effects
Study location					
Asia	5	5	369,125	1.09 (1–1.2); *I* ^2^ = 91.5%	Random effects
Europe	3	3	3061,232	0.95 (0.9–1); *I* ^2^ = 96.2%	Random effects
Type of AD					
AD	3	3	NA	1.05 (.95–1.16); *I* ^2^ = 92.4%	Random effects
Vascular dementia	3	3	NA	1.03 (.94–1.14); *I* ^2^ = 76.7%	Random effects
Others	3	4	NA	0.95 (.83–1.08); *I* ^2^ = 61.9%	Random effects
Age at dementia diagnosis					
50–59 years	2	2	NA	0.99 (.85–1.15); *I* ^2^ = 52.9%	Random effects
60–69 years	2	2	NA	1.01 (.92–1.11); *I* ^2^ = 69%	Random effects
≥70 years	2	2	NA	1.02 (.81–1.3); *I* ^2^ = 96.4%	Random effects
HZ involving cranial nerves	3	3	10,893^a^	1.36 (.97–2.43); *I* ^2^ = 92.5%	Random effects
Antiviral treatment					
Treated and untreated patients with VZV infection	3	3	358,542	0.74 (.68–0.8); *I* ^2^ = 39.8%	Fixed effects
Treated patients with and people without VZV infection	3	3	NA	0.91 (.9–0.93); *I* ^2^ = 0%	Fixed effects

Abbreviations: HZ, herpes zoster; VZV, varicella zoster virus.

^a^ No. of dementia case.

A meta‐analysis of just 8 of the studies with 3430,357 participants did not reveal a significant association between HZ and dementia incidence (OR: 1.05, 95% CI: .96–1.13; *p* = .28) (Table 2). When the studies were grouped according to study design, no significant association was observed in either case–control studies (OR: 0.99, 95% CI: .82–1.19; *p* = .54) or cohort studies (OR: 1.07, 95% CI: .97–1.17; *p* = .42). When studies were grouped according to location, no significant association was observed in studies from Asia (OR: 1.05, 95% CI: .98–1.14; *p* = .16) or Europe (OR: 0.95, 95% CI: .9–1; *p* = .056). In subgroup analysis according to type of dementia, HZ was not associated with Alzheimer's disease (OR: 1.05, 95% CI: .95–1.16; *p* = .35), vascular dementia (OR: 1.03, 95% CI: .94–1.14; *p* = .52), or other types of dementia (OR: 0.95, 95% CI: .83–1.08; *p* = .44). In terms of age at entry, patients with HZ aged 50–59 years (OR: 0.99, 95% CI: .85–1.15; *p* = .86), 60–69 years (OR: 1.01, 95% CI: .92–1.11; *p* = .83), or ≥70 years (OR: 1.02, 95% CI: .81–1.3; *p* = .84) did not have an increased risk for dementia.

In the assessment of the three studies that focused on HZ involving the cranial nerves, there was no significant association between HZ and dementia (OR: 1.36, 95% CI: .76–2.43; *p* = .304).

#### 3.3.2 Secondary analysis

**FIGURE 3:**
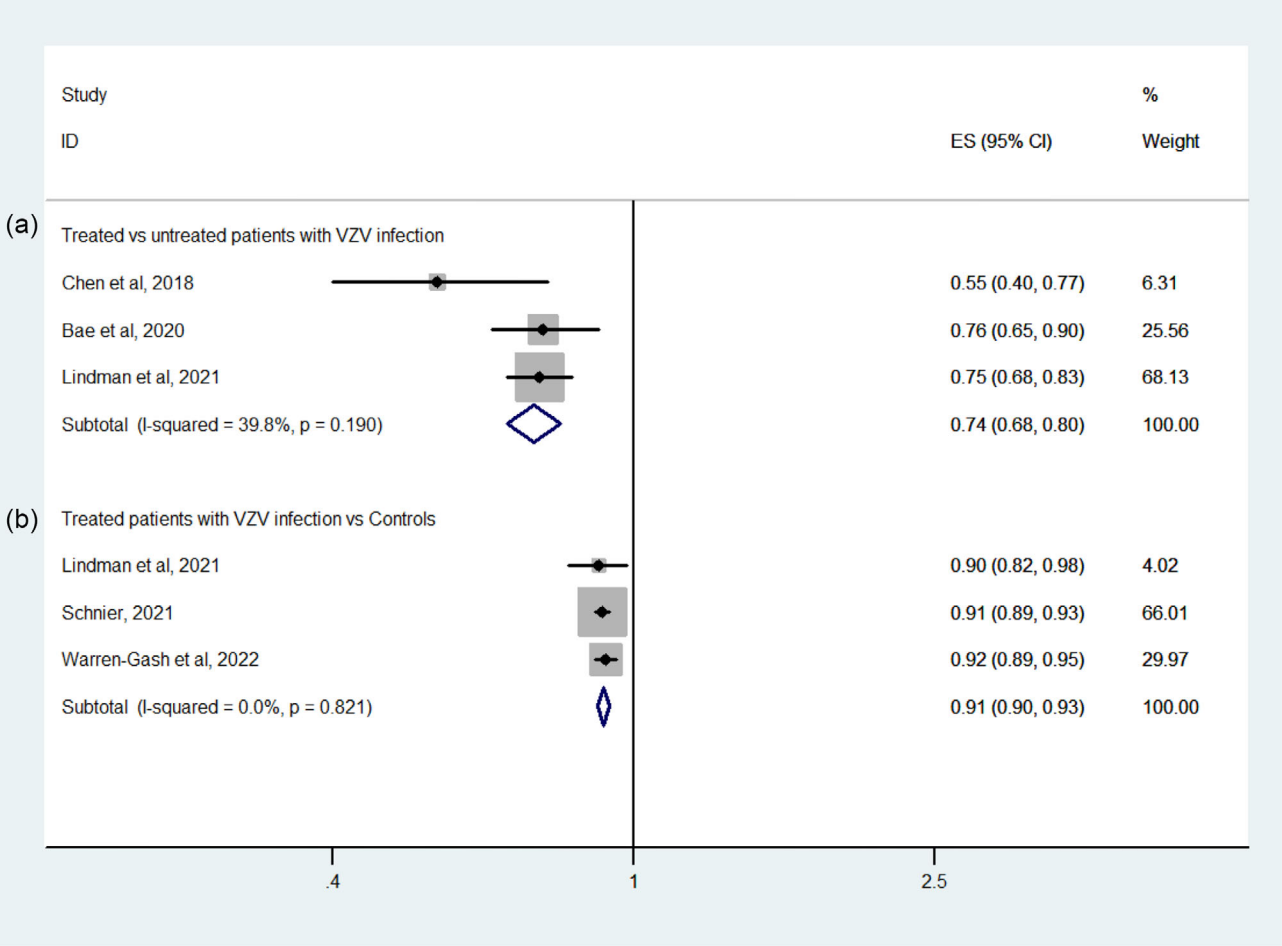
Forest plot of the risk of dementia in relation to antiviral treatment on patients with varicella zoster virus (VZV) infection: (A) treated versus untreated patients with VZV infection; (B) treated patients with VZV infection versus controls.

Three studies examined the effect of antiviral treatment on dementia by comparing treated and untreated patients infected with VZV; the pooled data indicated that patients who received antiviral treatment were less likely to develop dementia than those who did not (OR: 0.74, 95% CI: .68–0.8; *p* < .001) (Figure 3A). Three other studies evaluated the effect of antiviral treatment on dementia by comparing treated patients infected with VZV and people not infected with VZV; an analysis of the pooled data showed a lower dementia incidence among the former (OR: 0.91, 95% CI: .9−0.93; *p* < .001) (Figure 3B).

## 4 DISCUSSION

In our meta‐analysis of 10 population‐based studies, VZV infection or HZ was not associated with dementia. The results of subgroup analyses were consistent with this finding. In addition, no increased risk was apparent in patients with HZ of the cranial nerves. Notably, further analysis demonstrated that antiviral treatment was associated with a decreased risk for dementia. To the best of our knowledge, this is the first study to comprehensively synthesize all available population‐based research regarding the relationship among VZV infection, antiviral treatment, and dementia.

Many studies have explored the relationship between VZV infection and dementia because VZV can directly invade the brain and cause inflammation (Haanpää et al., 1998; Kinno et al., 2015). Previous studies reported viral replication in the cerebral arteries (Gilden et al., 2009; Stenmark et al., 2013). Consistent with those findings, VZV DNA was detected in the cerebrospinal fluid of patients with HZ who lacked central nervous system symptoms (Haanpää et al., 1998). VZV can invade the arterial walls to induce vasculopathy and subsequent dementia (Nagel et al., 2008). Virus reactivation–induced neuroinflammation is potentially involved. Inflammation has important roles in the etiologies of neurodegenerative disorders (Cooper et al., 2023; Zhang & Gao, 2022). One clinical study revealed inflammatory cells in the adventitia and intima of patients with both early and prolonged VZV infections (Nagel, 2014). Cytokines secreted by inflammatory cells may disrupt immune balance in the central nervous system. Previous studies claimed that the peripheral levels of interleukin‐6, transforming growth factor‐β1, and interleukin‐1α were useful early markers of dementia (Anuradha et al., 2022; Su et al., 2019). A meta‐analysis revealed that Alzheimer's disease was accompanied by inflammation of both the periphery and the cerebrospinal fluid (Swardfager et al., 2010).

However, we found no significant association between VZV infection and dementia. Considering the significant heterogeneity, we conducted further subgroup analyses. The incidence of dementia increases with age (Livingston et al., 2020). However, subgroup analysis according to age revealed no significant association in any age group. We suspect that the different types of dementia in these studies explain the presence of heterogeneity. Vascular dementia is caused by damage to brain–blood vessels or poor blood flow/oxygen delivery to the brain. A recent meta‐analysis of 12 studies showed that VZV infection was significantly associated with cerebrovascular and cardiovascular events (Erskine et al., 2017). Thus, it was possible that in patients infected with VZV, there is a higher risk of vascular dementia than Alzheimer's disease; however, surprisingly, this risk was not further investigated.

Our secondary analysis showed a significant association between treatment with antiviral agents and a lower risk for dementia, based on a comparison of treated and untreated patients infected with VZV; it may thus be the case that an association between VZV infection and dementia is masked by antiviral treatment. Even compared to controls, the incidence of dementia was significantly lower in patients infected with VZV who received antiviral treatment. Several possible mechanisms may explain the decreased risk for dementia with the use of antiviral agents. First, symptomatic HZ infection is treated using antiviral agents that relieve pain, accelerate skin lesion healing, and shorten the duration of virus shedding (Andrei & Snoeck, 2021). If treatment is initiated early, it may be possible to prevent the development of postherpetic neuralgia and other complications (Meyer et al., 2022; Vander Straten et al., 2001). A recent epidemiologic study demonstrated that antiviral therapy lowers the risk for HZ‐associated stroke (Kim et al., 2021), which is considered a risk factor for dementia. Second, although symptomatic HZ infection does not indicate an increased risk for dementia, the use of antiviral agents may alleviate subclinical VZV activity. Third, it should be noted that other herpes simplex viruses have been found to be associated with a higher risk for dementia (Cohen et al., 2023). Some antiviral agents used for VZV infection may therefore also be effective against other herpes viruses, such as herpes simplex virus 1. Further studies of the associations among VZV infection, antiviral treatment, and dementia should consider the status of other herpes virus infections.

The main strength of our study is that we performed detailed subgroup analyses according to study region and design, type of dementia, dementia measurement, age at entry, and classifications of VZV infection. Additionally, all studies were original, had high quality, and were adjusted for potential confounders.

However, our work had a few limitations, the most important of which was the small number of studies; this may have affected the results of subgroup analyses. Second, the clinical and statistical heterogeneities of the studies may have affected the robustness of our findings. Third, all studies were from Asia or Europe; the absence of work from the Americas or Africa may have affected the generalizability of our findings. Fourth, the severity of VZV infection may influence the risk for dementia. One of the included studies reported a higher risk in patients with a hospital record of VZV infection than in patients with a record of antiviral prescription. Fifth, our included studies lacked information on the effect of VZV vaccination on dementia risk. However, two larger studies have shown a rapid decline in the dementia rate after VZV vaccination (Eyting et al., 2023; Schnier et al., 2022), suggesting an important role of VZV in the etiology of dementia.

## 5 CONCLUSION

Our systematic review and meta‐analysis suggest that the null association between VZV infection and dementia may be masked by antiviral treatment. Additional high‐quality studies that consider antiviral therapy status and the severity of VZV infection are needed.

## AUTHOR CONTRIBUTIONS

Ting‐yun Ye and Li‐fei Zhu conceived the study and revised the manuscript critically for important intellectual content. Yu Mei and Min‐jing Zhu made substantial contributions to its design, acquisition, analysis, and interpretation of data. Min‐jing Zhu participated in the design, acquisition, analysis, and interpretation of data. All authors read and approved the final manuscript.

## CONFLICT OF INTEREST STATEMENT

The authors have no conflicts of interest to report.

## FUNDING INFORMATION

The authors have no funding to report.

### PEER REVIEW

The peer review history for this article is available at https://publons.com/publon/10.1002/brb3.3407.

## Supporting information


**Table S1** NOS for the assessment of quality of included studies: cohort studies.
**Table S2** NOS for assessment of quality of included studies: case–control studies.
**Figure S1** The Egger test for identifying publication bias in a meta‐analysis of observational studies.

## Data Availability

The data supporting the findings of this study are available within the article and/or its supplementary material.

## References

[brb33407-bib-0001] Andrei, G. , & Snoeck, R. (2021). Advances and perspectives in the management of varicella‐zoster virus infections. Molecules (Basel, Switzerland), 26(4), 1132. 33672709 10.3390/molecules26041132 PMC7924330

[brb33407-bib-0002] Anuradha, U. , Kumar, A. , & Singh, R. K. (2022). The clinical correlation of proinflammatory and anti‐inflammatory biomarkers with Alzheimer disease: A meta‐analysis. Neurological Sciences, 43, 285–298. 34032945 10.1007/s10072-021-05343-7

[brb33407-bib-0003] Bae, S. , Yun, S. C. , Kim, M. C. , Yoon, W. , Lim, J. S. , Lee, S. O. , Choi, S. H. , Kim, Y. S. , Woo, J. H. , Kim, S. Y. , & Kim, S. H. (2021). Association of herpes zoster with dementia and effect of antiviral therapy on dementia: A population‐based cohort study. European Archives of Psychiatry and Clinical Neuroscience, 271, 987–997. 32613564 10.1007/s00406-020-01157-4

[brb33407-bib-0004] Bangen, K. J. , Delano‐Wood, L. , Wierenga, C. E. , Stricker, N. H. , Hesselink, J. R. , & Bondi, M. W. (2010). Dementia following herpes zoster encephalitis. The Clinical Neuropsychologist, 24, 1193–1203. 20503134 10.1080/13854041003736778 PMC3013629

[brb33407-bib-0005] Chen, V. C. , Wu, S. I. , Huang, K. Y. , Yang, Y. H. , Kuo, T. Y. , Liang, H. Y. , Huang, K. L. , & Gossop, M. (2018). Herpes zoster and dementia: A nationwide population‐based cohort study. Journal of Clinical Psychiatry, 79, 16m11312. 10.4088/JCP.16m11312 29244265

[brb33407-bib-0006] Choi, H. G. , Park, B. J. , Lim, J. S. , Sim, S. Y. , Jung, Y. J. , & Lee, S. W. (2021). Herpes zoster does not increase the risk of neurodegenerative dementia: A case–control study. American Journal of Alzheimers Disease and Other Dementias, 36, 15333175211006504. 10.1177/15333175211006504 PMC11005322 33882722

[brb33407-bib-0007] Cohen, M. , Austin, E. , Bradu, S. , & Jagdeo, J. (2023). The association between herpes simplex virus and Alzheimer's disease: A systematic review. Journal of Drugs in Dermatology, 22, 1046–1052. 37801540 10.36849/JDD.6785

[brb33407-bib-0008] Cooper, J. , Pastorello, Y. , & Slevin, M. (2023). A meta‐analysis investigating the relationship between inflammation in autoimmune disease, elevated CRP, and the risk of dementia. Frontiers in Immunology, 14, 1087571. 36776896 10.3389/fimmu.2023.1087571 PMC9912841

[brb33407-bib-0009] Egger, M. , Davey Smith, G. , Schneider, M. , & Minder, C. (1997). Bias in meta‐analysis detected by a simple, graphical test. BMJ, 315, 629–634. 9310563 10.1136/bmj.315.7109.629 PMC2127453

[brb33407-bib-0010] Erskine, N. , Tran, H. , Levin, L. , Ulbricht, C. , Fingeroth, J. , Kiefe, C. , Goldberg, R. J. , & Singh, S. (2017). A systematic review and meta‐analysis on herpes zoster and the risk of cardiac and cerebrovascular events. PLoS ONE, 12, e0181565. 28749981 10.1371/journal.pone.0181565 PMC5531458

[brb33407-bib-0011] Eyting, M. , Xie, M. , Heß, S. , & Geldsetzer, P. (2023). Causal evidence that herpes zoster vaccination prevents a proportion of dementia cases. medRxiv. 2023 May, Preprint. 10.1101/2023.05.23.23290253

[brb33407-bib-0012] Gershon, A. A. , Breuer, J. , Cohen, J. I. , Cohrs, R. J. , Gershon, M. D. , Gilden, D. , Grose, C. , Hambleton, S. , Kennedy, P. G. , Oxman, M. N. , Seward, J. F. , & Yamanishi, K. (2015). Varicella zoster virus infection. Nature Reviews Disease Primers, 1, 15016. 10.1038/nrdp.2015.16 PMC5381807 27188665

[brb33407-bib-0013] Gilden, D. , Cohrs, R. J. , Mahalingam, R. , & Nagel, M. A. (2009). Varicella zoster virus vasculopathies: Diverse clinical manifestations, laboratory features, pathogenesis, and treatment. Lancet Neurology, 8, 731–740. 19608099 10.1016/S1474-4422(09)70134-6 PMC2814602

[brb33407-bib-0014] Grahn, A. , Nilsson, S. , Nordlund, A. , Lindén, T. , & Studahl, M. (2013). Cognitive impairment 3 years after neurological varicella‐zoster virus infection: A long‐term case control study. Journal of Neurology, 260, 2761–2769. 23900759 10.1007/s00415-013-7057-1

[brb33407-bib-0015] Haanpää, M. , Dastidar, P. , Weinberg, A. , Levin, M. , Miettinen, A. , Lapinlampi, A. , Laippala, P. , & Nurmikko, T. (1998). CSF and MRI findings in patients with acute herpes zoster. Neurology, 51, 1405–1411. 9818869 10.1212/wnl.51.5.1405

[brb33407-bib-0016] Heininger, U. , & Seward, J. F. (2006). Varicella. Lancet, 368, 1365–1376. 17046469 10.1016/S0140-6736(06)69561-5

[brb33407-bib-0017] Higgins, J. P. (2014). Cochrane handbook for systematic reviews of interventions version 5.1.0 . The Cochrane Collaboration. www.cochrane‐handbook.org

[brb33407-bib-0018] Higgins, J. P. , & Thompson, S. G. (2002). Quantifying heterogeneity in a meta‐analysis. Statistics in Medicine, 21, 1539–1558. 12111919 10.1002/sim.1186

[brb33407-bib-0019] Higgins, J. P. , Thompson, S. G. , Deeks, J. J. , & Altman, D. G. (2003). Measuring inconsistency in meta‐analyses. BMJ, 327, 557–560. 12958120 10.1136/bmj.327.7414.557 PMC192859

[brb33407-bib-0020] Johannesdottir Schmidt, S. A. , Veres, K. , Sorensen, H. T. , Obel, N. , & Henderson, V. W. (2022). Incident herpes zoster and risk of dementia: A population‐based Danish cohort study. Neurology, 99, e660–e668. 35676090 10.1212/WNL.0000000000200709 PMC9484607

[brb33407-bib-0021] Kim, J. , Jeon, J. , Lee, H. S. , & Lee, K. Y. (2021). Association between the risk for cardiovascular events and antiviral treatment for herpes zoster. Clinical Infectious Diseases, 73, 758–764. 32926085 10.1093/cid/ciaa1384

[brb33407-bib-0022] Kinno, R. , Kurokawa, S. , Uchiyama, M. , Sakae, Y. , Kasai, H. , Ogata, H. , & Kinugasa, E. (2015). False‐positive results obtained for immunoglobulin M antibody tests of cerebrospinal fluid for herpes simplex virus in a patient with varicella zoster virus encephalitis. Internal Medicine, 54, 2667–2670. 26466708 10.2169/internalmedicine.54.4891

[brb33407-bib-0023] Lau, J. , Ioannidis, J. P. , Terrin, N. , Schmid, C. H. , & Olkin, I. (2006). The case of the misleading funnel plot. BMJ, 333, 597–600. 16974018 10.1136/bmj.333.7568.597 PMC1570006

[brb33407-bib-0024] Livingston, G. , Huntley, J. , Sommerlad, A. , Ames, D. , Ballard, C. , Banerjee, S. , Brayne, C. , Burns, A. , Cohen‐Mansfield, J. , Cooper, C. , Costafreda, S. G. , Dias, A. , Fox, N. , Gitlin, L. N. , Howard, R. , Kales, H. C. , Kivimäki, M. , Larson, E. B. , Ogunniyi, A. , … Mukadam, N. (2020). Dementia prevention, intervention, and care: 2020 report of the Lancet Commission. Lancet, 396, 413–446. 32738937 10.1016/S0140-6736(20)30367-6 PMC7392084

[brb33407-bib-0025] Lopatko Lindman, K. , Hemmingsson, E. S. , Weidung, B. , Brännström, J. , Josefsson, M. , Olsson, J. , Elgh, F. , Nordström, P. , & Lövheim, H. (2021). Herpesvirus infections, antiviral treatment, and the risk of dementia‐a registry‐based cohort study in Sweden. Alzheimers Dement (N Y), 7, e12119. 33614892 10.1002/trc2.12119 PMC7882534

[brb33407-bib-0026] Lophatananon, A. , Mekli, K. , Cant, R. , Burns, A. , Dobson, C. , Itzhaki, R. , & Muir, K. (2021). Shingles, zostavax vaccination and risk of developing dementia: A nested case–control study‐results from the UK Biobank cohort. BMJ Open, 11, e045871. 10.1136/bmjopen-2020-045871 PMC8504358 34625411

[brb33407-bib-0027] Meyer, J. J. , Liu, K. , Danesh‐Meyer, H. V. , & Niederer, R. L. (2022). Prompt antiviral therapy is associated with lower risk of cerebrovascular accident following herpes zoster ophthalmicus. American Journal of Ophthalmology, 242, 215–220. 35809660 10.1016/j.ajo.2022.06.020

[brb33407-bib-0028] Nagel, M. A. (2014). Varicella zoster virus vasculopathy: Clinical features and pathogenesis. Journal of Neurovirology, 20, 157–163. 23918503 10.1007/s13365-013-0183-9 PMC3872206

[brb33407-bib-0029] Nagel, M. A. , Cohrs, R. J. , Mahalingam, R. , Wellish, M. C. , Forghani, B. , Schiller, A. , Safdieh, J. E. , Kamenkovich, E. , Ostrow, L. W. , Levy, M. , Greenberg, B. , Russman, A. N. , Katzan, I. , Gardner, C. J. , Häusler, M. , Nau, R. , Saraya, T. , Wada, H. , Goto, H. , … Gilden, D. H. (2008). The varicella zoster virus vasculopathies: Clinical, CSF, imaging, and virologic features. Neurology, 70, 853–860. 18332343 10.1212/01.wnl.0000304747.38502.e8 PMC2938740

[brb33407-bib-0030] Ou, Y. N. , Zhu, J. X. , Hou, X. H. , Shen, X. N. , Xu, W. , Dong, Q. , Tan, L. , & Yu, J. T. (2020). Associations of infectious agents with Alzheimer's disease: A systematic review and meta‐analysis. Journal of Alzheimer's Disease, 75, 299–309. 10.3233/JAD-191337 32280095

[brb33407-bib-0031] Schnier, C. , Janbek, J. , Lathe, R. , & Haas, J. (2022). Reduced dementia incidence after varicella zoster vaccination in Wales 2013–2020. Alzheimers Dement (N Y), 8, e12293. 35434253 10.1002/trc2.12293 PMC9006884

[brb33407-bib-0032] Schnier, C. , Janbek, J. , Williams, L. , Wilkinson, T. , Laursen, T. M. , Waldemar, G. , Richter, H. , Kostev, K. , Lathe, R. , & G Haas, J. (2021). Antiherpetic medication and incident dementia: Observational cohort studies in four countries. European Journal of Neurology, 28, 1840–1848. 33657269 10.1111/ene.14795

[brb33407-bib-0033] Shim, Y. , Park, M. , & Kim, J. (2022). Increased incidence of dementia following herpesvirus infection in the Korean population. Medicine, 101, e31116. 36254002 10.1097/MD.0000000000031116 PMC9575754

[brb33407-bib-0034] Steiner, I. , Kennedy, P. G. , & Pachner, A. R. (2007). The neurotropic herpes viruses: Herpes simplex and varicella‐zoster. Lancet Neurology, 6, 1015–1028. 17945155 10.1016/S1474-4422(07)70267-3

[brb33407-bib-0035] Stenmark, K. R. , Yeager, M. E. , El Kasmi, K. C. , Nozik‐Grayck, E. , Gerasimovskaya, E. V. , Li, M. , Riddle, S. R. , & Frid, M. G. (2013). The adventitia: Essential regulator of vascular wall structure and function. Annual Review of Physiology, 75, 23–47. 10.1146/annurev-physiol-030212-183802 PMC3762248 23216413

[brb33407-bib-0036] Su, C. , Zhao, K. , Xia, H. , & Xu, Y. (2019). Peripheral inflammatory biomarkers in Alzheimer's disease and mild cognitive impairment: A systematic review and meta‐analysis. Psychogeriatrics, 19, 300–309. 30790387 10.1111/psyg.12403

[brb33407-bib-0037] Swardfager, W. , Lanctôt, K. , Rothenburg, L. , Wong, A. , Cappell, J. , & Herrmann, N. (2010). A meta‐analysis of cytokines in Alzheimer's disease. Biological Psychiatry, 68, 930–941. 20692646 10.1016/j.biopsych.2010.06.012

[brb33407-bib-0038] Tsai, M. C. , Cheng, W. L. , Sheu, J. J. , Huang, C. C. , Shia, B. C. , Kao, L. T. , & Lin, H. C. (2017). Increased risk of dementia following herpes zoster ophthalmicus. PLoS ONE, 12, e0188490. 29166672 10.1371/journal.pone.0188490 PMC5699837

[brb33407-bib-0039] Vander Straten, M. , Carrasco, D. , Lee, P. , & Tyring, S. K. (2001). Reduction of postherpetic neuralgia in herpes zoster. Journal of Cutaneous Medicine and Surgery, 5, 409–416. 11907852 10.1007/s102270000024

[brb33407-bib-0040] Warren‐Gash, C. , Forbes, H. J. , Williamson, E. , Breuer, J. , Hayward, A. C. , Mavrodaris, A. , Ridha, B. H. , Rossor, M. N. , Thomas, S. L. , & Smeeth, L. (2019). Human herpesvirus infections and dementia or mild cognitive impairment: A systematic review and meta‐analysis. Scientific Reports, 9, 4743. 30894595 10.1038/s41598-019-41218-w PMC6426940

[brb33407-bib-0041] Warren‐Gash, C. , Williamson, E. , Shiekh, S. I. , Borjas‐Howard, J. , Pearce, N. , Breuer, J. M. , & Smeeth, L. (2022). No evidence that herpes zoster is associated with increased risk of dementia diagnosis. Annals of Clinical and Translational Neurology, 9, 363–374. 35170873 10.1002/acn3.51525 PMC8935278

[brb33407-bib-0042] Yawn, B. P. , & Gilden, D. (2013). The global epidemiology of herpes zoster. Neurology, 81, 928–930. 23999562 10.1212/WNL.0b013e3182a3516e PMC3885217

[brb33407-bib-0043] Zhang, P. F. , & Gao, F. (2022). Neuroinflammation in Parkinson's disease: A meta‐analysis of PET imaging studies. Journal of Neurology, 269, 2304–2314. 34724571 10.1007/s00415-021-10877-z

