# Supplementary material for: Effect of varicella‐zoster virus infection and antiviral treatment on the risk for dementia: A meta‐analysis of observational studies
Source: Brain Behav. 2024 Feb 5;14(2):e3407. doi: 10.1002/brb3.3407 (PMC10839536; doi:10.1002/brb3.3407)

| **Table S1 NOS for Assessment of Quality of Included Studies: Cohort Studies** | | | | | | | | |  |
| --- | --- | --- | --- | --- | --- | --- | --- | --- | --- |
| Study | Selection | | | | Comparability | | Outcomes | | |
|  | Representativeness of exposed cohort? | Selection of the nonexposed cohort? | Ascertainment of exposure? | Demonstration that outcome of interest was not represent at the start of the study | Comparability of Cohort* | Assessment of outcome | Was follow-up long enough for outcomes to occur | Adequacy of follow up of cohorts | |
| Tsai et al, 2017 | ★ | ★ | ★ | ★ | ★ | ★ | ★ | ★ | |
| Chen et al, 2018 | ★ | ★ | ★ | ★ | ★★ | ★ | ★ | ★ | |
| Bae et al, 2020 | ★ | ★ | ★ | ★ | ★★ | ★ | ★ | ★ | |
| Schmidt et al, 2022 | ★ | ★ | ★ | ★ | ★★ | ★ | ★ | ★ | |
| Shim et al, 2022 | ★ | ★ | ★ | ★ | ★★ | ★ | ★ | ★ | |
| Warren-Gash et al, 2022 | ★ | ★ | ★ | ★ | ★★ | ★ | ★ | — | |
| Lindman et al, 2021 | ★ | ★ | ★ | ★ | ★ | ★ | ★ | ★ | |
| Schnier, 2021 | ★ | ★ | ★ | ★ | ★ | ★ | ★ | ★ | |
| Note: A star denotes a score of 1; * A maximum of 2 stars can be allotted in this category | | | | | | | | | |

| **Table S2** NOS for Assessment of Quality of Included Studies: Case-Control Studies | | | | | | | | |
| --- | --- | --- | --- | --- | --- | --- | --- | --- |
| Study | Selection | | | | Comparability | Exposure | | |
|  | Is the case definition adequate | Representativeness of cases | Selection of controls | Definition of controls | Study controls for at least 3 additional factors | Ascertainment of exposure | Same method of ascertainment of exposure | Nonresponse rate |
| Choi et al, 2021 | ★ | **★** | ★ | ★ | — | **★** | ★ | — |
| Lophatananon et al, 2021 | ★ | **★** | ★ | ★ | ★★ | **★** | ★ | — |
| Note: A star denotes a score of 1; * A maximum of 2 stars can be allotted in this category | | | | | | | | |

**Figure S1** The Egger test for identifying publication bias in a meta-analysis of observational studies


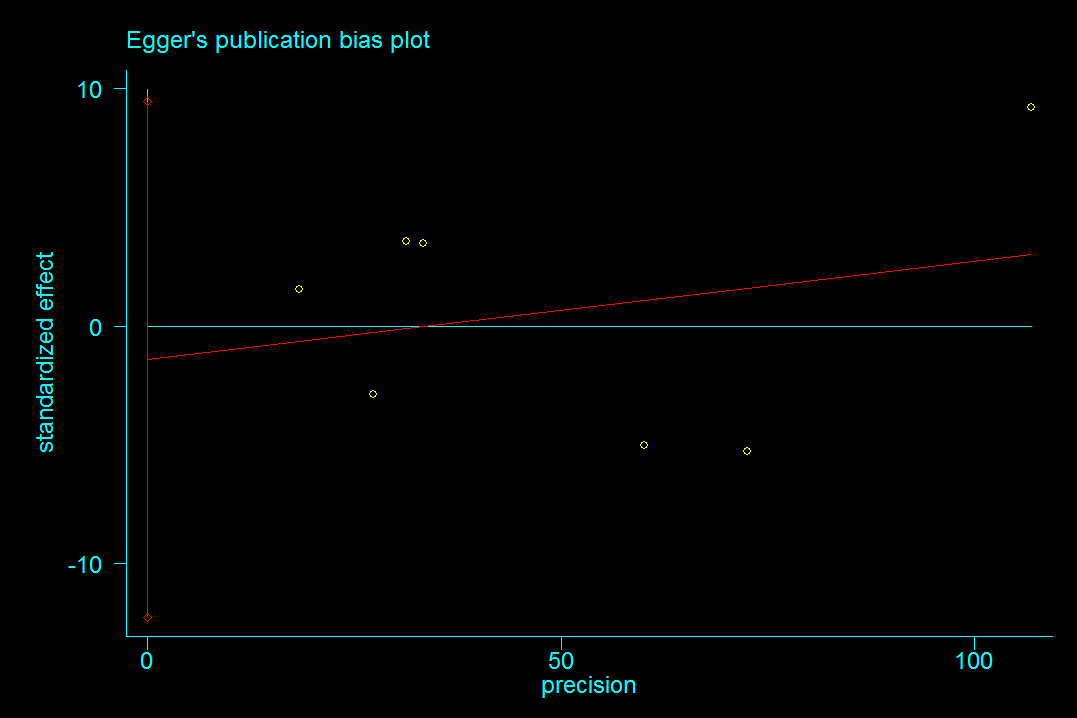

Supplement: Supplementary file 1 — Table S1 NOS for the assessment of quality of included studies: cohort studies. Table S2 NOS for assessment of quality of included studies: case–control studies. Figure S1 The Egger test for identifying publication bias in a meta‐analysis of observational studies. [file BRB3-14-e3407-s001.docx]
